# IL-31 plays dual roles in lung inflammation in an OVA-induced murine asthma model

**DOI:** 10.1242/bio.036244

**Published:** 2019-01-15

**Authors:** Junqiong Huang, Huan Yue, Tao Jiang, Jing Gao, Yu Shi, Bin Shi, Xiaoxue Wu, Xiaoqin Gou

**Affiliations:** 1 Medical Laboratory, Affiliated Hospital of Zunyi Medical University, Zunyi 563099, China; 2 School of Laboratory Medicine, Zunyi Medical University, Zunyi 563099, China; 3 Medical Laboratory, First People Hospital of Zunyi, Zunyi 563000, China; 4 Infectious Disease Department, First People Hospital of Zunyi, Zunyi 563000, China

**Keywords:** IL-31, Allergic asthma, Lung inflammation, Chemokine, CD4^+^ T cells

## Abstract

Interleukin 31 (IL-31) is a four-helix cytokine made predominantly by Th2 CD4^+^ T cells. It was initially identified as being associated with the promotion of atopic dermatitis, where increased levels of IL-31 levels have been found and IL-31 induced the expression of proinflammatory cytokines and chemokines in a human bronchial epithelial cell line. However, subsequent study has shown that IL-31RA knockout mice developed exacerbated type 2 inflammation in the lung following infection with *Schistosoma mansoni* eggs. In this study, we investigated the dynamic expression of IL-31 and IL-31RA during eight consecutive ovalbumin (OVA) challenges and measured the chemokines from lung alveolar epithelial cells induced by IL-31. In addition, we examined the effect deletion of IL-31RA has on lung inflammation and the differentiation of CD4^+^ T cells. Our results demonstrate that the expression of IL-31 and IL-31RA was elevated after each weekly OVA challenge, although slightly less of both observed after the first week of OVA challenge. IL-31 also promoted the expression of inflammatory chemokines CCL5, CCL6, CCL11, CCL16, CCL22, CCL28, CX3CL1, CXCL3, CXCL14 and CXCL16 in alveolar epithelial cells. Migration of macrophages and T cells was enhanced by culture supernatants of IL-31-stimulated alveolar epithelial cells. Lastly, and in contrast to the IL-31 results, mice deficient in IL-31RA developed exacerbated lung inflammation, increased IL-4-positive cell infiltrates and elevated Th2 cytokine responses in draining lymph nodes. The proliferation of IL-31RA^−/−^ CD4^+^ T cells was enhanced *in vitro* after anti-CD3/anti-CD28 antibody stimulation. These data indicate that IL-31/IL-31RA may play dual roles, first as an early inflammatory mediator promoting the secretion of chemokines to recruit inflammatory cells, and subsequently as a late inflammatory suppressor, limiting Th2 cytokine responses in allergic asthma.

## Abstract (precis)

**Summary:** IL-31 plays dual roles, acting as an early inflammatory mediator promoting the secretion of chemokines to recruit inflammatory cells and subsequent negative feedback pathway for suppressing the magnitude of type 2 inflammation.

## INTRODUCTION

Interleukin 31 (IL-31) is a four-helix bundle cytokine structurally related to IL-6 and produced, in particular, by Th2 CD4^+^ T cells (Dillon et al., 2004). Studies carried out in mouse models as well as data obtained from the analysis of sera and biopsies from patients with atopic dermatitis (AD) indicate that IL-31 is involved in the pathogenesis of allergic skin inflammatory diseases and is upregulated in these diseases (Neis et al., 2006; Siniewicz-Luzenczyk et al., 2013; Bilsborough et al., 2006; Raap et al., 2008; Kato et al., 2014). More recently IL-31 was found at increased levels in sera of patients with allergic asthma (Lei et al., 2008; Chai et al., 2017; Lai et al., 2016). In addition, the receptor for IL-31 was enhanced in both lung tissue and lung cellular infiltrates from a mouse model of airway hyper-responsiveness (Dillon et al., 2004). Also, IL-31 alone or in combination with IL4 or IL-13 could elevate the expression of epidermal growth factor (EGF), vascular endothelial growth factor (VEGF) and monocyte chemoattractant protein-1 (MCP-1/CCL2) from human bronchial epithelial BEAS-2B cells, which contribute to airway inflammation (Ip et al., 2007). Moreover, IL-31 has been shown to induce chemotaxis and release of CCL26 in eosinophils, which are known to play an important role in the pathogenesis of allergic diseases (Kunsleben et al., 2015; Larose et al., 2015; Provost et al., 2013).

The IL-31 heterodimeric receptor is composed of IL-31RA (gp130-like receptor/GPL or IL-31Rα) and oncostatin M receptor (OSMR) (Dillon et al., 2004). IL-31RA contributes indirectly by cytokine binding and recruitment of signal-transducing partner of OSMR, which leads to intracellular phosphorylation and activation of the Jak1, Jak2, STAT-1, STAT-3, STAT-5, p38 MAPK, ERK1/2, PI3K/Akt and JNK signaling pathways (Diveu et al., 2004; Chattopadhyay et al., 2007; Dambacher et al., 2007). It was reported that SOCS (suppressor of cytokine signaling) 1, SOCS2 and SOCS3 increased upon IL-31 treatment and IL-31-induced STAT3 activation is strongly inhibited by SOCS3 (Maier et al., 2015). IL-31 heterodimeric receptors are abundantly expressed in many tissues including skin, lung, intestine, spleen, brain, bone marrow, thymus and testis, and epithelial cells (Dillon et al., 2004; Dambacher et al., 2007; Jawa et al., 2008). Expression of IL-31 receptor combined with the diversity of function of IL-6 family members supports that IL-31 may have distinct roles in different conditions. Interestingly, two studies carried out on IL-31 receptor A knockout (KO) (IL-31RA^−/−^) mice showed that IL-31 negatively regulated Th2 cytokine-dependent immunity and inflammation. After intravenous injection of *Schistosoma mansoni* eggs, IL-31RA^−/−^ mice developed exacerbated pulmonary granulomatous inflammation and had higher levels of IL-4, IL-5 and IL-13 in lymph node cells compared to wild-type (WT) counterparts. IL-31RA^−/−^ CD4^+^ T cells exhibited enhanced proliferation and expressed elevated levels of IL-4 and IL-13 messenger RNA *in vitro* under neutral stimulation condition with anti-CD3/anti-CD28 (Perrigoue et al., 2007). The authors also demonstrated that IL-31R^−/−^ mice exhibit enhanced intestinal inflammation and Th2 cytokine responses following Trichuris infection (Perrigoue et al., 2009). These are somewhat contrary to the theory that IL-31 plays an active role in the development and exacerbation of the Th2-associated disease.

In contrast, Bilsborough et al. reported that mice deficient in IL-31RA exhibited increased responsiveness to OSM (oncostatin M) and enhanced production of OSM-inducible cytokines, such as IL-6 and VEGF, during airway sensitization and challenge, suggesting that susceptibility of IL-31RA^−/−^ mice to exacerbated Th2-type diseases is an indirect result of IL-31RA deletion that leads to an elevated responsiveness to OSM (Bilsborough et al., 2010). However, in this study neutralization of OSM has been found to have a limited effect in decreasing OSM, IL-6, VEGF and tissue inhibitor of metalloproteinases 1 *(*TIMP-1) in bronchoalveolar lavage fluid (BALF) after anti-OSM treatment. A possible explanation for these apparently contradictory roles of IL-31 might depend on tissue-specific cell types expressing IL-31RA. For example, IL-31 receptor signaling positively regulates Th2 responses induced by nasal administration of the Japanese cedar pollen allergen, but negatively regulates these responses following intraperitoneal injection (Saito et al., 2017). In an attempt to further understand the role of IL-31 in modulating airway inflammation, we investigated the dynamic levels of IL-31 in a mouse model of allergic asthma, as well as the effect of IL-31 on lung alveolar epithelial cells. The effects of IL-31R signaling on airway inflammation and the proliferation and differentiation of CD4^+^ T subsets were explored using IL-31RA KO mice.

## RESULTS

### Expression of IL-31 and IL-31R was elevated in asthma model mice

**Fig. 1.**
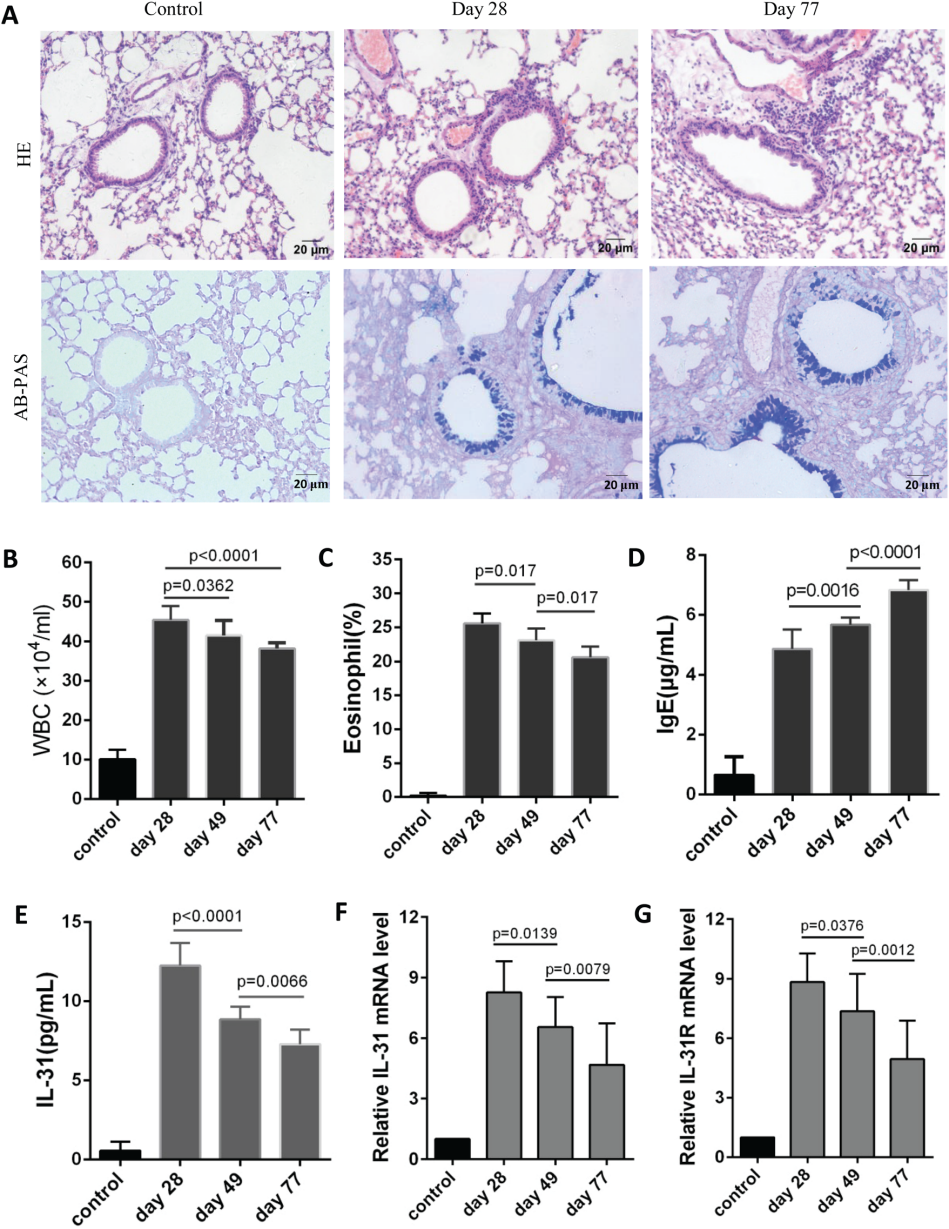
**IL-31 and IL-31R mRNA expression was upregulated in the asthma mouse model.** On days 0, 7 and 14 the mice were injected intraperitoneally with 100 μg OVA, and 5% OVA was delivered by pump atomization on day 21 for 8 consecutive weeks (30 min/day, 3 days/week). Within 24 h after the last atomization, the mice were euthanized, and lung tissue, BALF and peripheral blood were collected for analysis of histopathology, infiltrates, IgE levels and the expression of IL-31 and IL-31RA. (A) Lungs were stained with HE and AB-PAS. (B,C) Cell infiltrates in BALF (*n*=10). (D) IgE levels in peripheral blood (*n*=10). (E) IL-31 levels in peripheral blood (*n*=10). (F,G) IL-31 and IL-31R mNA expression in lung tissues was detected by fluorescent quantitative PCR (*n*=10). Results expressed as the mean±s.d. Statistical significance was determined by two-tailed one-way ANOVA analysis for multiple comparisons after data from each group were proved to fit normal distribution. The experiment was repeated twice.

To explore the dynamic changes in IL-31 and IL-31RA *in vivo*, we established an asthma mouse model. Mice were sensitized with OVA on days 0, 7 and 14 and challenged with the same antigen (Ag) at day 21 for 8 consecutive weeks. Notable inflammatory cell infiltrates around the airways and blood vessels were observed in all allergic asthma models. Increases in goblet cell and mucus in airway were also found (Fig. 1A). Infiltrates in BALF were higher than control group but gradually decreased at days 49 and 77 compared with day 28 (Fig. 1B,C), whereas IgE levels in peripheral blood were elevated gradually (Fig. 1D). Consistent with infiltrates in BALF, the levels of IL-31 in blood were significantly higher after the initial atomization than those in the control, with a gradual decline at days 49 and 77 (Fig. 1E). IL-31RA, a novel type I cytokine receptor, mediates IL-31 signaling when coupled with OSMR, which is expressed ubiquitously. We collected lung tissue after OVA challenge for analysis of IL-31 and IL-31RA mRNA. IL-31RA mRNA was upregulated in OVA-challenged mice consistent with Dillon et al., (2004), and was abated at days 49 and 77 as did BALF infiltrates (Fig. 1G). The similar trend was detected in expression of IL-31 mRNA in lungs (Fig. 1F), suggesting that IL-31 may affect lung epithelial cells.

### IL-31 induced chemokine expression in alveolar epithelial cells

**Fig. 2.**
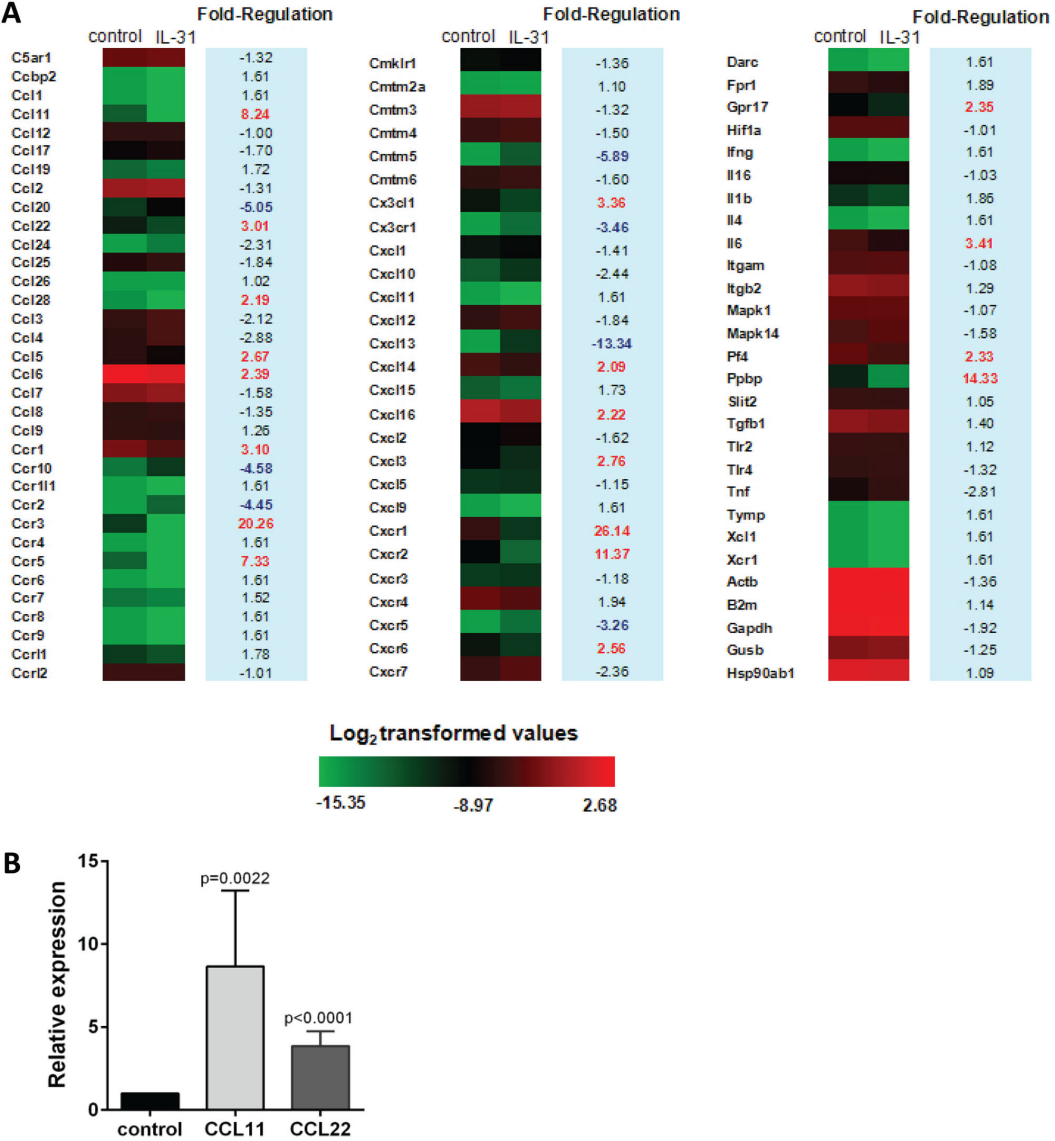
**IL-31 induced chemokine expression in mouse alveolar epithelial cells.** Alveolar epithelial cells were treated with 100 ng/ml IL-31 for 24 h and total RNA was extracted for chemokine expression. Differential gene expression between group treated with IL-31 and control group was calculated by 2^−ΔΔCt^. (A) Chemokine expression in alveolar epithelial cells was analyzed by cDNA microarray. (B) CCL11 and CCL22 expression were detected by fluorescent quantitative PCR (*n*=6). Data were shown as the mean±s.d. Significance was determined by two-tailed Student's *t*-test after data from each group were proved to fit normal distribution.

IL-31 stimulates proinflammatory cytokines and chemokines in monocytes, macrophages, dendritic cells, keratinocytes and bronchial epithelial cells (Kasraie et al., 2011; Kasraie et al., 2010; Horejs-Hoeck et al., 2012). We investigated the effect of IL-31 on the expression of inflammatory cytokines and chemokines in mouse alveolar epithelial cells *in vitro*. After 24 h stimulation of alveolar epithelial cells (purity: 95.1%) with IL-31, total RNA was extracted for analysis of chemokine expression using gene array chip technology. We found a large number of gene differences between the group treated with IL-31 and the control group, with 19 genes being upregulated more than twofold in IL-31-treated cells. Some of these upregulated genes are critical for the migration of eosinophil, T cell and macrophage, such as CCL5, CCL6, CCL11, CCL16, CCL22, CCL28 and CX3CL1, which are linked to the airway inflammation (Coelho et al., 2007; Shaw et al., 2017; English et al., 2006; Isgro et al., 2013; Chang et al., 2012). Transcripts for CXCL3, CXCL14 and CXCL16 were elevated, related to the recruitment of neutrophils and dentritic cells (Engl et al., 2004; Woehrl et al., 2010). The increase of some chemokine receptors, such as CCR1, CCR3, CCR5, CXCR1, CXCR2 and CXCR6, and proinflammatory factor IL-6 was detected (Fig. 2A). Further fluorescent quantitative PCR analysis confirmed that CCL11 and CCL22 expression was increased in mouse alveolar epithelial cells following IL-31 stimulation (Fig. 2B).

### Migration was enhanced by supernatants from alveolar epithelial cells treated with IL-31

**Fig. 3.**
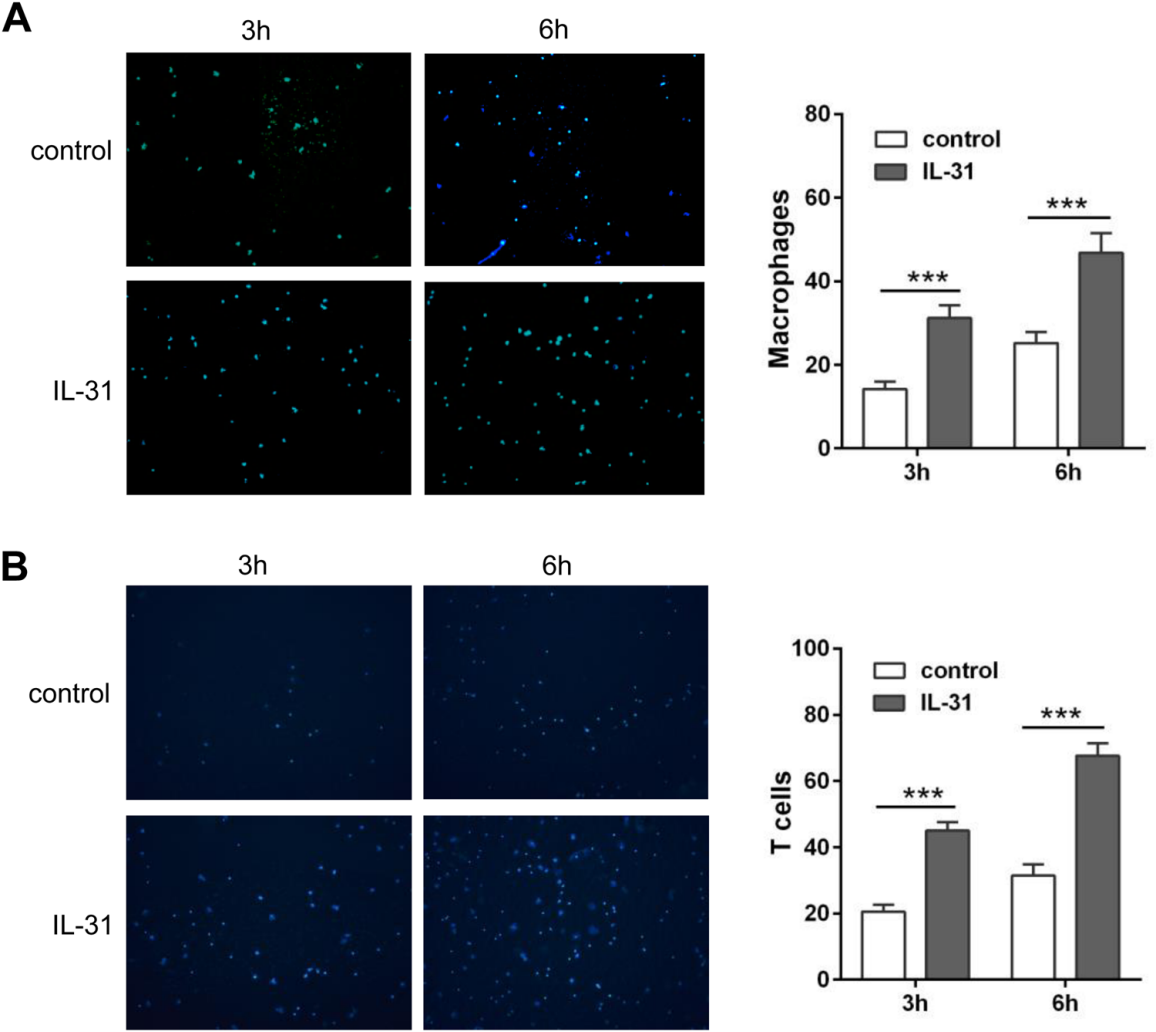
**Cell migration was enhanced by culture media supernatant from IL-31-stimulated alveolar epithelial cells.** Alveolar epithelial cells were treated with 100 ng/ml recombinant IL-31 for 24 h. Culture media supernatant was added to the lower chamber of Transwell plates and cell suspensions of macrophages or T lymphocytes was added to the upper chamber. Migrated cells were counted under a fluorescence microscope at 3 h and 6 h, respectively. Culture media from IL-31-stimulated cells induced greater cell migration than the controls. (A) Macrophages (*n*=5). (B) T lymphocytes (*n*=5). Shown is the mean (±s.d.) of five replicates per condition at each time point for one experiment. Significance was determined by two-tailed Student's *t*-tests for two groups. ***P<0.001. The experiment was replicated twice.

Inflammatory cells such as eosinophils, lymphocytes and macrophages, are recruited to lung in the presence of chemokines in asthma. We demonstrated that the expression of chemokines related to recruitment of macrophages and T cells was increased in alveolar epithelial cells upon stimulation with IL-31. To explore the chemotactic effect of the chemokines secreted by alveolar epithelial cells, we performed chemotaxis *in vitro* by Transwell migration assay. Supernatants from alveolar epithelial cells treated with IL-31 were collected and added to the lower chamber to recruit macrophages (purity: 90.2%) and T lymphocytes (purity: 96.5%) plated in the upper chamber. For both macrophages (Fig. 3A) and T cells (Fig. 3B), higher cell migration was detected in the group treated with culture supernatants from alveolar epithelial in time-dependent manner, compared with the control group. This indicates that IL-31 may be involved in recruitment of macrophages and T cells through induction of chemokine secretion in lung epithelial cells, which is important for maintenance of inflammatory infiltrates.

### IL-31RA KO mice developed exacerbated airway inflammation after OVA challenge

**Fig. 4.**
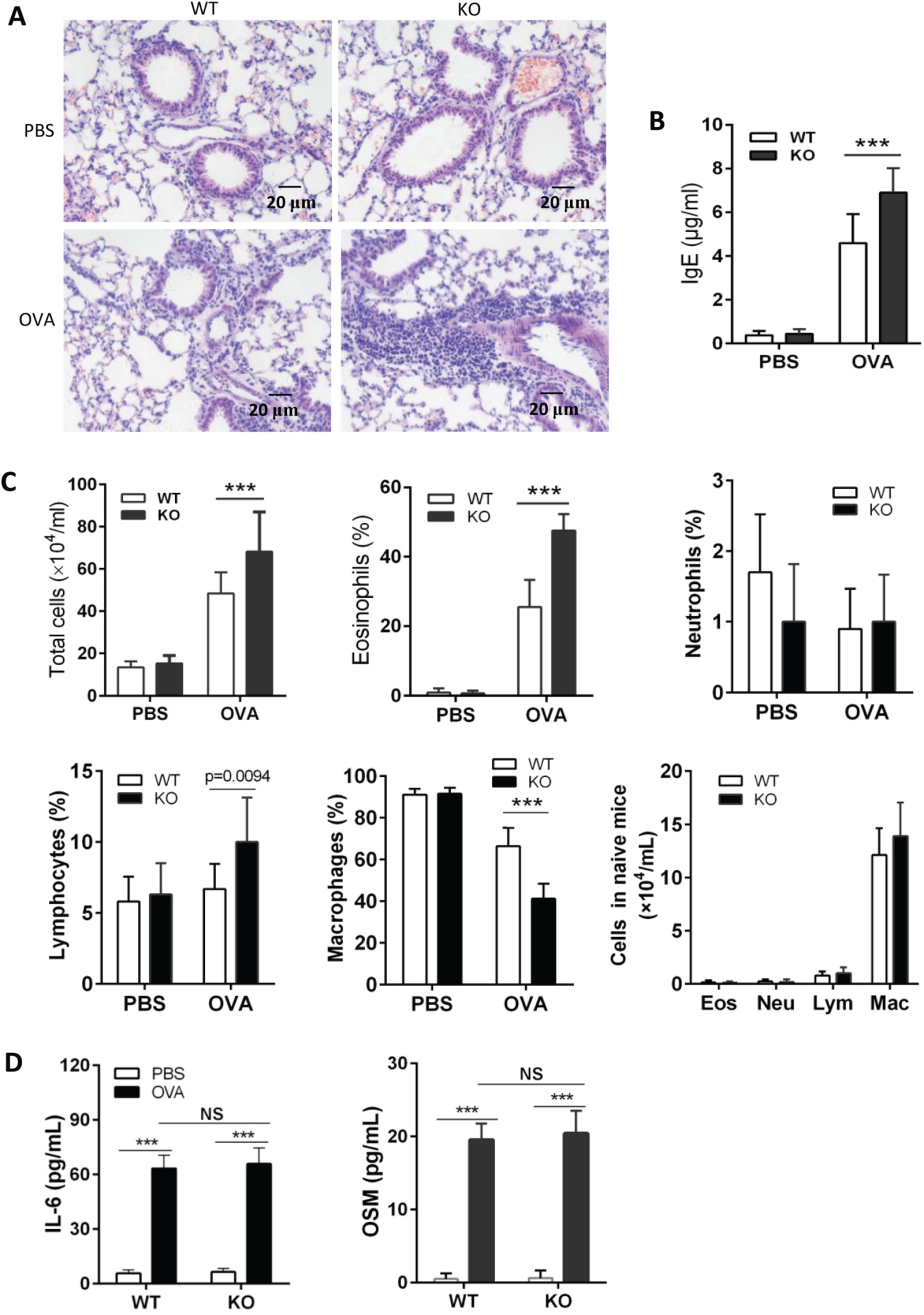
**IL-31RA KO mice exhibit exacerbated lung inflammation following challenge with OVA.** IL-31RA KO mice were generated to delete the fourth exon of IL-31RA by homologous recombination. Ten IL-31RA KO mice were sensitized intraperitoneally with 100 μg OVA in the presence of aluminum hydroxide at days 0, 7 and 14, and an intranasal challenge with 5% OVA started on day 21 for 7 consecutive days. (A) Paraffin sections of lungs from OVA challenged mice were HE stained. (B) Serum was assayed for total IgE (*n*=10). (C) BALF was collected for analysis of cell infiltrates (*n*=10). (D) IL-6 and OSM in BALF were detected by ELISA (*n*=8). Data expressed as the mean±s.d. Significance was determined by two-tailed Student's *t*-test, ***P<0.001. The experiment was repeated twice. Eos, eosinophil; Lym, lymphocyte; Neu, neutrophil; Mac, macrophage.

To determine the potential regulatory function of IL-31–IL-31R interaction in lung inflammation in an allergic asthma model, IL-31RA KO mice were generated by homologous recombination and demonstrated to be deficient in coding exon 4 of α chain of IL-31 receptor. Any substantial difference was not found between WT and IL-31RA KO mice through analysis of peripheral blood cells and histopathology of lung, spleen and thymus (Jiang et al., 2015). After OVA sensitization and challenge, IL-31RA KO mice showed more inflammatory infiltrates as compared to WT counterparts (Fig. 4A). Higher IgE levels in peripheral blood (Fig. 4B) and more infiltrates in BALF (Fig. 4C) were also detected in IL-31RA KO mice than those in WT mice after intranasal OVA challenge. This is consistent with the finding that mice deficient in IL-31RA develop exacerbated inflammation in lung after *Schistosoma mansoni* egg injection (Perrigoue et al., 2007). Interestingly, no difference in inflammation infiltrates in BALF between WT and IL-31RA KO mice treated with PBS (Fig. 4C, lower right graph), which is inconsistent with the finding that IL-31RA KO mice had significantly increased percentages of neutrophils and lymphocytes compared with WT mice (Bilsborough et al., 2010). Since IL-31 shares signaling overlap with OSM and IL-6, levels of IL-6 and OSM in BALF were measured by ELISA after OVA sensitization and challenge. No difference was found in levels of IL-6 and OSM between WT and IL-31RA KO mice (Fig. 4D).

### Th2 cytokine response was enhanced in IL-31RA KO mice

**Fig. 5.**
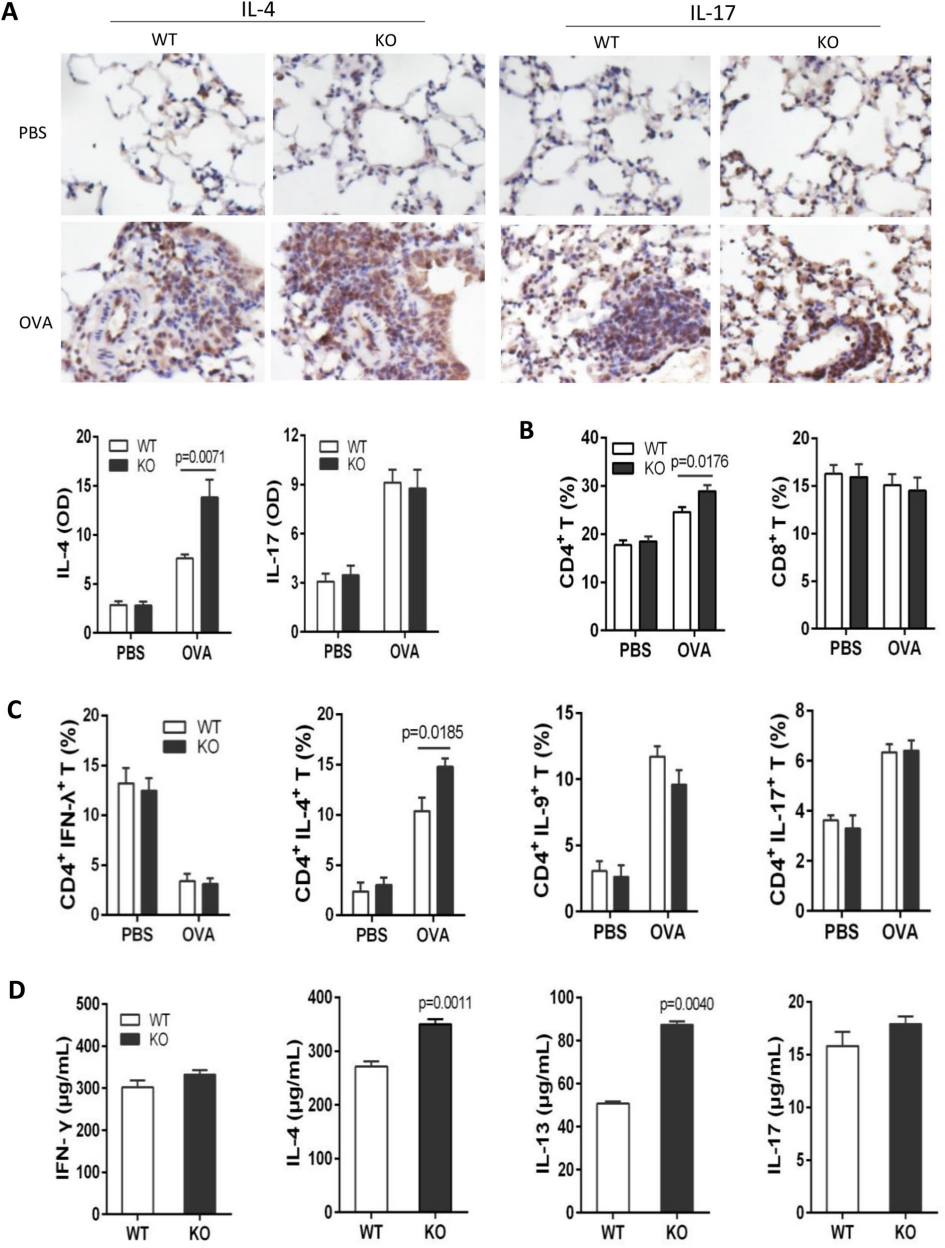
**Enhanced Th2 cytokine responses in the draining lymph nodes following challenge with OVA.** Ten IL-31RA KO mice were sensitized intraperitoneally with 100 ug OVA in the presence of aluminum hydroxide at days 0, 7 and 14, and an intranasal challenge with 5% OVA started on day 21 for 7 consecutive days. (A) Lungs were collected for detection of Th2 and Th17 infiltrates by immunohistochemistry (*n*=6). (B) CD4^+^ T cells and (C) Th1, Th2, Th9 and Th17 populations in peripheral blood were analyzed by flow cytometry (*n*=5–10). (D) Draining lymph node cells from OVA-challenged mice were stimulated with 20 μg/ml of OVA for 48 h. Levels of IFN-γ, IL-4, IL-13 and IL-17 in supernatants detected by ELISA (*n*=5-6). Data expressed as the mean±s.e.m. Significance was determined by two-tailed Student's *t*-test. The experiment was repeated twice.

Previous reports have demonstrated that draining lymph node cells from mice lacking IL-31RA exhibited higher levels of IL-4, IL-5 and IL-13 following restimulation with *S. mansoni* eggs. To determine whether Th2 responses are influenced in IL-31RA KO mice during allergic airway inflammation, Th2 and Th17 infiltrates in lungs were detected after the last atomization by histoimmunochemistry assay using anti-IL-4 and anti-IL17 antibodies, and optical density (OD) was measured using ImageProPlus 6.0 software. More IL-4-positive cells in lungs were observed in OVA-challenged IL-31RA KO mice compared with WT counterparts, whereas no difference in IL-17-positive cells was detected between WT and IL-31RA KO mice (Fig. 5A). Consistent with lung histochemistry, CD4^+^ T cells were significantly higher in peripheral blood in mice deficient in IL-31RA than those in WT counterparts (Fig. 5B). An increase in percentage of CD4^+^ IL-4^+^ T cells, but not CD4^+^ IFN-γ^+^, CD4^+^ IL-9^+^ and CD4^+^ IL-17^+^ T cells, was found between WT and IL-31RA KO mice (Fig. 5C). Draining lymph nodes cells were isolated from OVA-challenged WT or IL-31RA KO mice and restimulated with the same antigen. After restimulation with OVA, Draining lymph node cells isolated from OVA-challenged IL-31RA KO mice produced higher levels of IL-4 and IL-13 following antigen-specific restimulation (Fig. 5D).

### IL-31R signaling suppressed the proliferation of CD4^+^ T cells

**Fig. 6.**
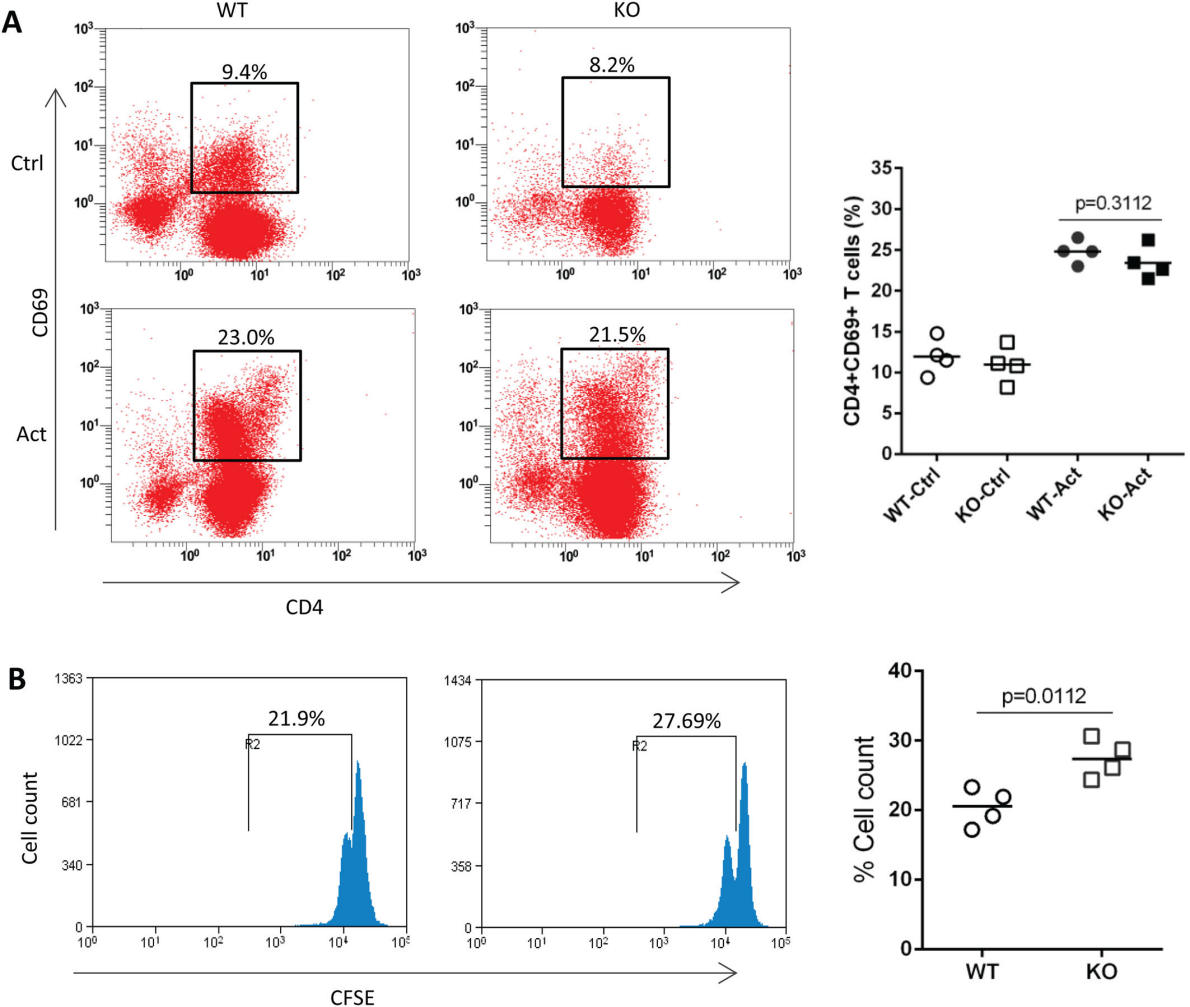
**IL-31R signaling suppressed the proliferation of CD4^+^ T cells.** Purified CD4^+^ T cells from WT and IL-31RA KO mice were stimulated with anti-CD3/anti-CD28. The activation (*n*=4) (A) and proliferation (*n*=4) (B) of CD4^+^ T cells were assayed by flow cytometry. All values are plotted and lines indicate means. Statistical significance was determined by two-tailed Student's *t*- tests. The experiment was repeated twice. Ctrl, control; Act, activation.

The increase of Th2 infiltrates in lungs and IL-4 levels secreted by antigen-specific draining lymph node cells suggested an enhanced Th2 response in OVA-challenged IL-31RA KO mice. To determine whether IL-31RA signaling influences the activation and proliferation of CD4^+^ T cells, spleen naïve CD4^+^ T cells were isolated from WT and IL-31RA KO mice and stimulated with anti-CD3/anti-CD28. No difference in the percentage of CD69-positive T cells was found between WT and IL-31RA KO mice following anti-CD3/anti-CD28 stimulation (Fig. 6A). CFSE staining was performed before 4 days of co-culture with anti-CD3/anti-CD28 to address the question of whether the elevated Th2 response was associated with an increase in T cell proliferation. The proportion of CFSE-positive IL-31RA^−/−^ CD4^+^ T cells was found to be increased compared to that of WT CD4^+^ T cells (Fig. 6B), indicating a higher proportion of division of IL-31RA^−/−^ CD4^+^ T cells.

### IL-31R signaling did not influence the differentiation of Th sub-populations

**Fig. 7.**
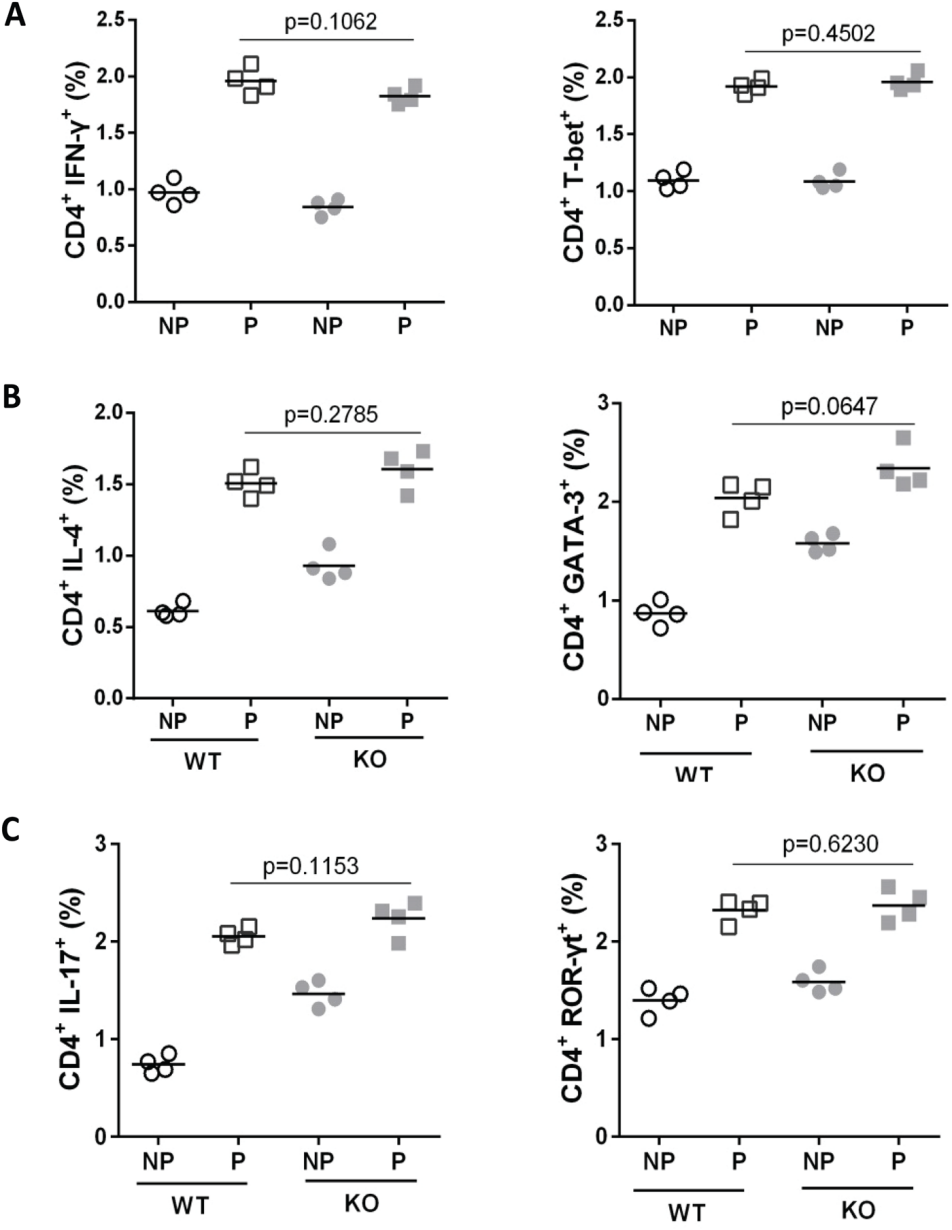
**IL-31R signaling did not influence the differentiation of CD4^+^ T cell sub-populations.** Purified CD4^+^ T cells from WT and IL-31RA KO mice were stimulated with anti-CD3/anti-CD28 under Th1-, Th2- or Th17- polarizing conditions. Cells were assayed for the transcription factors T-bet, GATA-3, ROR-γt and cytokines IFN-γ, IL-4, IL-17 by flow cytometry. No difference on the differentiation of Th1(A), Th2 (B) and Th17 (C) was found between WT (*n*=4) and IL-31RA KO mice (*n*=4). All values are plotted and lines indicate means. Statistical significance was determined by two-tailed Student's *t*- tests. The experiment was repeated three times. P, polarization; NP, non-polarization.

To investigate whether IL-31RA signaling drives CD4^+^ T cell differentiating toward Th2, naïve CD4^+^ T cells were isolated from WT and IL-31RA KO mice and stimulated with anti-CD3/anti-CD28 under neutral and Th1-, Th2- or Th17- polarizing condition. Three days after stimulation, no difference in the expression of IL-4 or the transcription factor GATA-3 was observed between WT and IL-31RA KO mice under Th2-polarizing condition (Fig. 7B), indicating that IL-31RA signaling does not regulate Th2 differentiation. This phenomenon was also found in cell differentiation under Th1- and Th17-polarizing conditions, with similar expression of Th1 and Th17 type cytokine and transcription factors between WT and IL-31RA KO mice (Fig. 7A,C). Combined with the increase in Th2 infiltrates in lungs, these data suggest that IL-31 may influence Th2 type inflammation by regulating proliferation and/or recruitment of CD4^+^ T cells rather than differentiation of CD4^+^ T subsets.

## DISCUSSION

In this study, we first investigated IL-31 levels in peripheral blood and expression of IL-31RA in the lungs of mice undergoing allergic airway inflammation following 8 consecutive weeks of intranasal challenge with OVA. The results demonstrated that higher levels of IL-31 in peripheral blood and elevated IL-31 mRNA in lungs persistently existed during 2 months of airway inflammation, although with a slight decrease at later time-points as compared with those at day 28, indicating that IL-31 modulated the development of airway inflammation in asthma. It has been reported that an increase in IL-31 levels was detected in patients with allergic rhinitis and IL-31 induced production of IL-4, IL-5 and IL-13 in PBMC and nasal epithelial cells from patients with allergic rhinitis (Stott et al., 2013). Serum IL-31 levels were significantly elevated in patients with asthma and correlated positively with Th2 type cytokines IL-5 and IL-13, asthma severity or total serum IgE (Lai et al., 2016). Previous studies demonstrated that IL-31 induced the production of inflammatory cytokines and chemokines in human primary keratinocytes, monocytes/macrophages and bronchial epithelial cells, which was enhanced by IL-4 and IL-13 (Ip et al., 2007; Kasraie et al., 2011; Kasraie et al., 2010). These data suggest that IL-31 may positively regulate the inflammation of Th2 type diseases via induction of chemokines, which recruit infiltrates in local tissue.

We also found that IL-31RA mRNA was elevated in lungs throughout 8 consecutive weeks of allergic airway inflammation following OVA challenge as compared with PBS control, indicating consecutive elevated IL-31R signaling in lungs. It has been reported that IL-31 induced expression of chemokines contributing to airway inflammation and tissue damage in human bronchial epithelial BEAS-2B cells (Ip et al., 2007). To determine whether IL-31 stimulates the production of inflammatory cytokines and chemokines in lung epithelial cells, type II alveolar epithelial cells isolated from mice were stimulated with recombinant mouse IL-31 and RNA was extracted for analysis of cytokines and chemokines by gene array chip. An increase in expression of 19 gene was found in IL-31-treated cells, some of which are related to the migration of eosinophils, T cells and monocytes/macrophages, including CCL5, CCL6, CCL11, CCL16, CCL22, CCL28 and CX3CL1 (Isgro et al., 2013; Rose et al., 2010; Matsukura et al., 2010; Viney et al., 2014; Ooi et al., 2012). Consistent with this, we also found that chemokine receptors mRNA, such as CCR1, CCR3, CCR5, CXCR1, CXCR2 and CXCR6, were increased in alveolar epithelial cells after IL-31 stimulation. In addition, the migration of macrophages and T cells was increased by supernatants of alveolar cells stimulated with IL-31 in a time-dependent manner. These data demonstrated that IL-31 promoted lung inflammation in allergic asthma via induction of chemokines in alveolar epithelial cells. However, anti-IL-31 antibodies did not neutralize the production of Th2 type cytokines either *in vitro* or *in vivo* mouse models of allergic asthma (Bilsborough et al., 2010). One explanation is that IL-31 is one of these Th2-type cytokines that induce inflammation-related cytokines, such as IL-4, IL-5 and IL-13, which form the complex network of cytokines that governs the development allergic asthma and so that role of IL-31 may be covered by other stronger cytokines.

IL-31RA expression has been detected on a wide range of cell types including macrophages, mast cells, T cells, and epithelial cells (Dillon et al., 2004; Diveu et al., 2004; Dreuw et al., 2004; Yamaoka et al., 2009) and IL-31 is produced by activated CD4^+^ T cells, eosinophils, macrophages and dendritic cells (Dillon et al., 2004; Kunsleben et al., 2015; Cornelissen et al., 2011). Loss of IL-31R signaling in IL-31RA KO mice resulted in exacerbated inflammation, increased IgE levels and IL-4-positive infiltrates, and more IL-4 and IL-13 secreted by draining lymph nodes cells after OVA sensitization and challenge, indicating an enhanced Th2 response in antigen-challenged IL-31RA KO mice. This is consistent with the findings that parasites induced exacerbated Th2 type inflammation in lung and intestines of mice deficient in IL-31RA, and CD4^+^ T cells from IL-31RA KO mice exhibited enhanced proliferation (Perrigoue et al., 2007; Perrigoue et al., 2009). However, Bilsborough et al. (Bilsborough et al., 2010) reported that IL-31 receptor KO mice exhibited elevated responsiveness to oncostatin M. They also found that IL-31RA KO mice had significantly increased percentages of neutrophil and lymphocyte populations before OVA stimulation, compared with WT mice. In contrast, in our study, no increase in infiltrates was detected in BALF collected from naïve mice deficient in IL-31RA. However, we did observe enhanced proliferation of CD4^+^ T cells in IL-31RA KO mice, whereas no difference in the activation of CD4^+^ T cells was found between WT and IL-31RA KO mice. Th2 and recently reported Th17 subsets have been implicated in the regulation of many immune responses linked to lung inflammation in patients with asthma (Zhou et al., 2017). To address the question of whether IL-31 influences the differentiation of Th2 and Th17, naïve CD4^+^ T cells were stimulated with anti-CD3/anti-CD28 under neutral or Th1-, Th2-, Th17- polarizing condition. No difference in the expression of cytokines IFN-γ, IL-4, IL-17 and transcription factors T-bet, GATA-3, ROR-γt in CD4^+^ T cells was observed between naïve WT and IL-31RA KO mice after stimulation of CD4^+^ T cells with anti-CD3/anti-CD28 under neutral or Th1-, Th2-, Th17- polarizing conditions. These data demonstrate that IL-31-IL-31R interaction negatively regulates lung inflammation in asthma through induction of proliferation of CD4^+^ T cell and production of Th2 effector cytokines, not promotion of CD4^+^ T cell subset differentiation.

Most cytokines have the ability to mediate pleiotropic effects, depending on the cytokine milieu and tissues in which their receptors are expressed (Hunter and Kastelein, 2012; Fontes et al., 2015; Tamasauskiene and Sitkauskiene, 2017; Fisher et al., 2014). It has become clear that many IL-6 family cytokines suppress inflammatory responses, although many that signal through gp130 have critical proinflammatory effects (Chen et al., 2017; Pyle et al., 2017; Su et al., 2016). IL-27 can promote Th1 responses via augmenting proliferation and secretion of IFN-γ by naïve CD4^+^ T cells (Yoshimoto et al., 2007; Pflanz et al., 2002; Chen et al., 2000). However, IL-27ra^−/−^ mice infected with *Toxoplasma gondii* generated an IFN-γ response to limit parasite replication, and subsequently developed lethal CD4^+^ T cell mediated inflammation (Artis et al., 2004a; Artis et al., 2004b; Batten et al., 2006; Miyazaki et al., 2005; Stumhofer et al., 2006). IL-31RA is a novel member of the gp130-subfamily, sharing the conserved structural motifs of type I cytokine receptor and mediates IL-31 signaling when coupled with OSMR (Dillon et al., 2004; Diveu et al., 2004). IL-31 binding with the IL-31RA/OSMRB complex activates the JAK/STAT signaling pathway, which involves STAT1, STAT3 and STAT5 phosphorylation (Dambacher et al., 2007; Kasraie et al., 2013). STAT3 activation by IL-31 induces the expression of SOCS3, which inhibits further IL-31 signaling in a negative feedback loop, through inhibition of JAK activity (Maier et al., 2015). As a novel member of IL-6 family, IL-31 may have dual roles due to the complex of its heterodimer receptor and cytokine milieu, acting as an early proinflammatory regulator and subsequent negative feedback pathway for suppressing the magnitude of type 2 inflammation.

In summary, we first demonstrated that IL-31 promoted lung inflammation in allergic asthma mice via inducing production of chemokines in alveolar epithelial cells to recruit cell infiltrates. Our results from an asthma model using IL-31RA KO mice showed that IL-31R signaling negatively regulates inflammation through suppressing the proliferation of CD4^+^ T cells, which led to the decreased production of Th2-type cytokines. These data indicated that IL-31 may play dual roles, first an early proinflammation Th2 response followed by a later negative feedback response, associated with allergic asthma. Although our study demonstrated that IL-31-IL-31R interaction does not influence the differentiation of Th1, Th2 and Th17, the role of IL-31 in suppression or induction of Treg responses remains to be examined. Moreover, while much of the early work has attempted to identify the function of IL-31 in isolation, it is likely that the effects of IL-31R signaling are regulated by other pro- and anti-inflammatory cytokines.

## MATERIALS AND METHODS

### Animals

Six to 8 week-old WT C57BL/6 and BALB/c mice were purchase from Center of Experiment Animal of Third Military Medical University. Mice were bred in a specific pathogen-free environment at the Zunyi Medical University. Mice were acclimatized for 2–3 weeks before performing experiments. IL-31RA KO mice were generated at Lexicon Genetics as previously described (Jiang et al., 2015). In brief, a targeting vector was constructed to delete of coding exon 4 of α chain of IL-31 receptor (IL-31RA) by homologous recombination with LacZNeo selectable marker cassette. The linearized recombinant vector pKOS-53-LacZNeo was electroporated into 129/SvEvBrd-derived embryonic stem (ES) cells and subjected to positive selection with G418. Chimeras (IL-31RA^+/−^) were generated by injection of targeted ES cells in C57BL/6 blastocysts and transplantation into pseudopregnant females. Male chimeras were crossed with germ-line heterozygous females to generate offspring deficient in IL-31RA (IL-31RA^−/−^). The progeny were subsequently identified by PCR genotyping strategy. Three primers, WT-specific sense primer (5'-GGGTGTGAACGCTGGAATAATG-3′), KO-specific sense primer (5'-GCAGCGCATCGCCTTCTATC-3′) and a common antisense primer (5'-GATCATCTCTCCAAATTTACATG-3′), were used in multiplex PCR reaction. This primer-triplet amplifies a 274 bp fragment of the WT allele and a 348 bp fragment of the mutant allele. Analysis of IL-31RA mRNA and protein expression was performed using RT-PCR and western blot to confirm deletion of IL-31RA in lung of homozygous IL-31RA KO mice versus their heterozygous and WT littermates. According to the criteria pre-established, 8–10 week-old (20–25 g) mice were included in this study. Mice were assigned to each group by random computer allocation. All experiments were performed following the guidelines of the Ethics Committee of Zunyi Medical University.

### Mouse model of allergic asthma

On days 0, 7 and 14, mice were injected intraperitoneally with 0.2 ml OVA (Sigma-Aldrich) suspension [100 μg OVA was dissolved in 0.1 ml saline and mixed with equal volume of aluminum hydroxide (Sigma-Aldrich)]. The mice received 5% OVA by pump atomization at day 21 for 7 consecutive days (30 min/day) or 8 weeks (30 min/day, 3 days/week). At days 28, 35, 49, 63 and 77, the animals were euthanized and BALF was collected to determine infiltrate cells by two investigators who were blind to the groups. Peripheral blood was harvested for analysis of IL-31 and IgE levels by ELISA.

### Culture of draining lymph node cells

Single cell suspensions of draining lymph nodes from WT and IL-31RA KO mice sensitized and challenged with OVA for 7 consecutive days were plated at 2 million cells/ml in complete RPMI 1640 media and stimulated with either media alone or 20 ug/mL OVA. After 48 h of culture, cells were harvested and analyzed for CD4^+^IL-4^+^, CD4^+^IFN-γ^+^, CD4^+^IL-9^+^ and CD4^+^IL-17^+^ T cell populations by flow cytometry (Beckman Coulter). Cell culture supernatants were also collected after 48 h and IFN-γ, IL-4, IL-13 and IL-17 levels were measured by ELISA.

### Detection of cytokines and IgE

Levels of IL-31, IL-6, OSM and total IgE in peripheral blood or BALF were measured using ELISA kits (R&D Systems). Levels of IFN-γ, IL-4, IL-13 and IL-17 in supernatants were detected using standard sandwich ELISA protocols (eBioscience). Quantification was performed by regression analysis from a standard curve generated from molecule standards included in the kits.

### Culture of type II alveolar epithelial cells (AECs)

After mice were euthanized, lungs were exposed, perfused and lavaged. Lungs were filled with a mixture of 0.25% trypsin (HyClone) and 0.1% collagenase (Sigma-Aldrich) (1:1 mixture) for 20 min at 37°C and then dissected. Dissected lungs were then treated with DNase I (Invitrogen) for 5 min and serially filtered through 70 μm, 40 μm, and 20 μm filters (Merck). The crude suspensions were cultured in Petri dishes coated with IgG (Boster) for 3 h at room temperature. The non-adherent cells were then collected by centrifugation at 150 ***g*** for 6 min. The purity of AECs was determined by immunocytochemistry using rabbit anti-mouse SP-A antibodies (CAT#sc-13977, Santa Cruz) and goat anti-rabbit IgG-FITC (CAT#BA1105, Boster) at 1:100 dilution. For induction of chemokines, AECs were plated into six-well culture plates. When the cultures reached a density of 70%, the cells were incubated first in serum-free medium for 2 h and then changed to medium containing recombinant mouse IL-31 (100 ng/ml) (PeproTech). The control cells were treated with PBS. After 24 h, the cells were harvested for analysis of gene array chip and real-time PCR.

### Isolation of T cells and macrophages

The spleens from C57BL/6 mice were minced and fibrous material was removed by filtration through a 70 μm cell strainer (BD Falcon). Erythrocytes were lysed with NH_4_Cl solution (Tiangen) and T cells were purified on nylon wool columns (Ye et al., 2008), and followed by negative selection using MagCellect Mouse CD3^+^ T Cell Isolation Kits (R&D Systems). Cells were collected and stained with anti-CD3-FITC (CAT#85-11-0032-82, eBioscience) at 4°C for 30 min and analyzed by flow cytometry. For isolation of peritoneal macrophages, mice were injected intraperitoneally with RPMI 1640 (HyClone), and cells were harvested from the peritoneal cavity 10 min after injection. The cells were washed with phosphate-buffered saline (PBS), plated, and allowed to adhere at 37°C for 2 h in Dulbecco's Modified Eagle's Medium (DMEM; HyClone). At the end of that period, non-adherent cells were removed and macrophages were harvested. The purity of macrophages was determined by flow cytometry using anti-CD14-PE (CAT#12-0141, eBioscience).

### Chemotaxis assay *in vitro*

Mouse T cells and macrophages were cultured in RPMI 1640 media supplemented with 10% FBS for chemotaxis assays. Supernatants from mouse alveolar epithelial cells treated with 100 ng/ml recombinant mouse IL-31 were added to the lower chamber of the Transwell culture plate (Corning). Macrophage or T lymphocyte suspensions (1 million cells/ml) were added to the upper chamber. The plates were cultured at 37°C under 5% CO_2_ for 3 h and 6 h. The polycarbonate membrane was then removed and a cotton swab was used to remove cells that had not migrated from the filter top. The migrated cells on the lower side of membrane were stained with DAPI (Boster) and their number was determined by two independent observers who were blind to the groups by counting five random fields per well using an fluorescence microscopy (Carestream Health).

### T cell proliferation

Spleen CD4^+^ T cells were isolated from WT and IL-31RA KO mice by negative selection via incubation with hybridoma supernatants (αB220, αFCγ, αCD8, αMHCII) followed by magnetic bead purification (Life Technologies). T cells at 2 million cells/ml were washed and resuspended in 2 μM CFSE (Invitrogen) staining solution for 10 min, and CFSE fluorescence was measured by flow cytometry at 96 h after culture with 1 μl/ml anti-CD3 (CAT#85-16-0032-81)/anti-CD28 (CAT#85-16-0281-81) (both eBioscience).

### T cell activation and differentiation

CD4^+^ T cells were resuspended at 2 million cells/ml and stimulated with anti-CD3/anti-CD28 (1 μl/ml) under neutral or Th1- (50 ng/ml IL-12, 125 ng/ml anti-IL-4), Th2- (40 ng/ml IL-4, 50 ng/ml anti-IFN-γ), Th17- (50 ng/ml IL-6, 2 ng/ml TGF-β1) polarizing conditions. At 72 h, T cells were stimulated by phorbol myristate acetate (PMA) and brefeldin A (BFA) (Sigma-Aldrich) for 6 h. Subsequently, cells were stained with anti-CD4-FITC (CAT#85-11-0041-82), anti-IFN-γ-PE (CAT#85-12-7311-82), anti-IL-4-APC (CAT#85-17-7041-82), anti-IL-17A-PE (CAT#85-12-7177-81), anti-T-bet-PE (CAT#85-12-5825-80), anti-GATA-3-PE (CAT#85-12-9966-41) and anti-ROR-γt-PE (CAT#85-12-6981-80) (all eBioscience) at 4°C for 30 min and analyzed by flow cytometry. For activation assays, CD4^+^ T cells were treated with anti-CD3/anti-CD28 antibodies for 48 h. Cells were harvested and stained with anti-CD69-PE (CAT#85-12-0691-81, eBioscience) at 4°C for 30 min, and then analyzed by flow cytometry.

### Real-time PCR

Real-time PCR was used for analysis of the expression of IL-31 and IL-31RA in lungs, and the expression of CCL11 and CCL22 in cell culture. Total RNA was extracted using Trizol (Invitrogen). cDNA was generated per standard protocol with Prime Script™ reverse transcriptase (Takara) and used as input for real-time PCR. Taqman PCR reactions were run at standard conditions and analyzed using a BIO-RAD C1000 PCR machine (BIO-RAD). Differential expression of mRNA was analyzed using β-actin (Invitrogen) as an internal reference and relative expression of target genes in both experimental and control samples was calculated.

### Gene array chip analysis

Alveolar epithelial cells were treated with 100 ng/ml recombinant mouse IL-31 for 24 h and total RNA was extracted as described previously. The expression of chemokines and their receptors was analyzed using gene array chip technology. Fluorescent quantitative PCR was performed as follows: denaturation at 95°C for 10 min, followed by 40 cycles of denaturation at 95°C for 15 s, annealing and elongation at 60°C for 1 min, and melting at 55°C–95°C for 10 s. Differential gene expression (upregulated, fold change ≥2; downregulated, ≤-2) between alveolar epithelial cells activated by IL-31 and control cells was calculated by the 2^−ΔΔCt^ method.

### Histopathology

Lung tissues were fixed in formalin and embedded in paraffin. Paraffin sections were deparaffinized to washing. In routine Hematoxylin and Eosin (HE) staining, sections were treated with Hematoxylin for 5 min, Eosin for 2 min, and mounted by neutral gum. For Alcian Blue-Periodic Acid Schiff (AB-PAS) staining, paraffin sections were stained with AB dye for 10 min, periodic acid oxidation for 15 min, and Schiff liquid for 20 min, and followed by dehydration, transparent and mounting. Histopathology of the lung tissues was examined under an optical microscope BX51 (Olympus).

### Immunohistochemistry

Immunohistochemistry for IL-4 and IL-17 was performed on 4 µm-thick lung tissue sections. Antigen on slides was retrieved with 1 mM EDTA (Boster) and slides were pretreated with 3% H_2_O_2_ (Boster) to quench endogenous peroxidase activity. After being blocked with normal goat serum, slides were incubated with anti-IL-4 (CAT#bs-0581-R) and anti-IL-17 (CAT#bs-1183-R) monoclonal antibodies (both Bioss) diluted 1:100 overnight at 4°C and treated with biotin-conjugated goat anti-rabbit IgG antibodies (CAT#bs-0295G-R, Bioss) diluted 1:50 for 30 min at room temperature. Slides were then stained with horseradish peroxidase (HRP)-conjugated streptavidin for 30 min and immunoperoxidase staining was developed using a 3,3′diaminobenzidine (DAB) for 5 min. IL-4- and IL-17-positive cells were examined using a BX-51 microscope and optical density (OD) was measured using ImageProPlus 6.0 software.

### Statistical analysis

Statistical analysis was performed with GraphPad Prism version 6.02. Student's *t*-test was used to test for statistical significance when comparing data between two groups in normal distribution. Multiple comparisons were performed using one-way or two-way ANOVA. For all statistical tests, a two-tailed *P*-value <0.05 was regarded statistically significant.
