# Correction: IL-31 plays dual roles in lung inflammation in an OVA-induced murine asthma model

**DOI:** 10.1242/bio.059869

**Published:** 2023-04-03

**Authors:** Junqiong Huang, Huan Yue, Tao Jiang, Jing Gao, Yu Shi, Bin Shi, Xiaoxue Wu, Xiaoqin Gou

There was an error published in *Biol. Open* (2019) **8**, bio036244 (doi:10.1242/bio.036244).

In Fig. 4, the knockout (KO) PBS image in panel A was erroneously a duplication of the wild-type PBS image. A replicate knockout panel has been substituted. The corrected Fig. 4 is shown below.

**Fig. 4.**
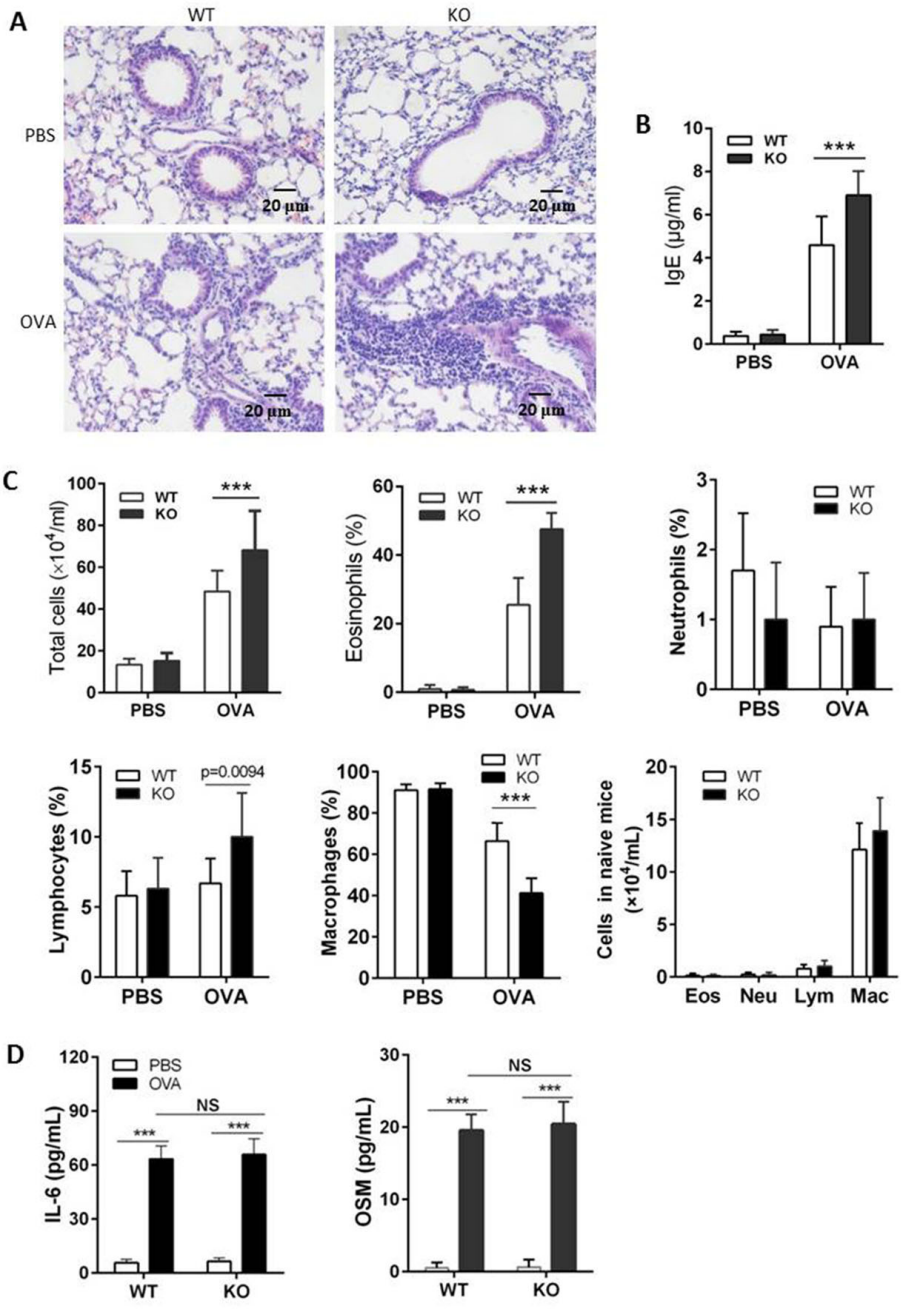
**IL-31RA KO mice exhibit exacerbated lung inflammation following challenge with OVA.** IL-31RA KO mice were generated to delete the fourth exon of IL-31RA by homologous recombination. Ten IL-31RA KO mice were sensitized intraperitoneally with 100 μg OVA in the presence of aluminum hydroxide at days 0, 7 and 14, and an intranasal challenge with 5% OVA started on day 21 for 7 consecutive days. (A) Paraffin sections of lungs from OVA challenged mice were HE stained. (B) Serum was assayed for total IgE (n=10). (C) BALF was collected for analysis of cell infiltrates (n=10). (D) IL-6 and OSM in BALF were detected by ELISA (n=8). Data expressed as the mean ±s.d. Significance was determined by two-tailed Student's t-test, ***P<0.001. The experiment was repeated twice. Eos, eosinophil; Lym, lymphocyte; Neu, neutrophil; Mac, macrophage.

The authors apologise to readers for this error, which does not impact the results or conclusions of this paper.

